# NEUROMYODredger: Whole Exome Sequencing for the Diagnosis of Neurodevelopmental and Neuromuscular Disorders in Seven Countries

**DOI:** 10.1111/cge.14736

**Published:** 2025-02-25

**Authors:** Edoardo Malfatti, Alexandru Caramizaru, Hane Lee, JiHye Kim, Hussein Shoaito, Alessandra Pennisi, Sarah Souvannanorath, François‐Jérôme Authier, Andreea Dumitrescu, Nagia Fahmy, Rosa Elena Escobar‐Cedillo, Antonio Miranda‐Duarte, Alexandra Berenice Luna‐Angulo, Sonia Nouioua, Ouissem Benchaabi, Meriem Tazir, Sihem Hallal, Peggy Martinez, Claudia Castiglioni, Amelia Dobrescu, Homa Tajsharghi

**Affiliations:** ^1^ Reference Center for Neuromuscular Disorders APHP Henri Mondor University Hospital France; ^2^ University Paris Est Créteil, Inserm, U955, IMRB Créteil France; ^3^ Regional Center for Medical Genetics Dolj Craiova Romania; ^4^ Department of Medical Genetics University of Medicine and Pharmacy of Craiova Craiova Romania; ^5^ 3billion Inc. Seoul South Korea; ^6^ Pediatrics and Continuing Care Departement Victor Dupouy Hospital Argenteuil France; ^7^ Neuromuscular Center, Neuropsychiatry Department, Faculty of Medicine Ain Shams University Cairo Egypt; ^8^ Instituto Nacional de Rehabilitación Luis Guillermo Ibarra Ibarra Ciudad de México Mexico; ^9^ Department of Neurology Cherchell EHS Tipaza Algeria; ^10^ NeuroSciences Research Laboratory University of Algiers Benyoucef Benkhedda Algiers Algeria; ^11^ Central Laboratory of Biochemistry CHU Mustapha Algiers Algeria; ^12^ Neurofisiología clínica Instituto Nacional de Salud del Niño San Borja Lima Peru; ^13^ Departamento de Neurologia Clinica MEDS‐INRPAC Santiago Chile; ^14^ School of Health Sciences, Division of Biomedicine University of Skovde Skovde Sweden

**Keywords:** international consortium, low‐income countries, myopathies, neurodevelopmental disorders, neuromuscular disorders, next generation sequencing, rare disorders, whole exome sequencing

## Abstract

Although substantial advancements have been made in genetic testing, several barriers continue to limit patient access, leading to delays in diagnosis, effective treatments, and preventative measures. The NEUROMYODredger‐3billion Megaproject End the Diagnostic Odyssey grant offered free whole exome sequencing (WES) to 245 patients with undiagnosed neurodevelopmental or neuromuscular disorders in seven countries: Algeria, Chile, Egypt, France, Mexico, Peru, and Romania. We found pathogenic variants in 79 patients (diagnostic yield 32.24%)—36 neurodevelopmental (43.90%) and 43 neuromuscular (26.38%). Fifty patients harboured variants of uncertain significance (VUS, 20.40%)—14 neurodevelopmental (17.07%) and 36 neuromuscular (22.08%), and 116 patients had negative results (47.34%). NEUROMYODredger helped end the diagnostic odyssey in around 30% of patients, while ongoing functional studies and reanalysis strategies are used in order to reach more diagnoses. In conclusion, a singleton WES approach is valuable in determining the genetic diagnosis of neurodevelopmental and neuromuscular diseases, especially in low and middle‐income countries.

## Graphical abstract

NEUROMYODredger‐3billion Megaproject was created to provide a genetic diagnosis for 245 children and adults with neurologic disorders in seven countries for free. With whole exome sequencing on a single patient, we could end the diagnostic odyssey in around 30% of cases.

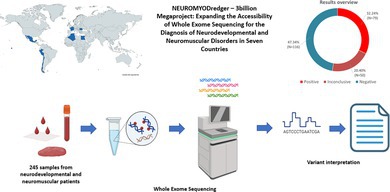

## 1 Introduction

Neurodevelopmental and neuromuscular disorders are two clinically and genetically heterogeneous groups of disorders, often representing a diagnostic challenge for physicians, as well as for patients and their families [1, 2]. According to DSM‐5 (Diagnostic and Statistical Manual of Mental Disorders 5), a neurodevelopmental disorder (NDD) involves a developmental period onset deficit that leads to a neurological functioning impairment, often having a genetic component [3]. In neuromuscular disorders (NMDs), progressive muscle degeneration and weakness are the common manifestations, and many entities have a genetic cause [4, 5].

Although affected individuals have benefited from the general evolution and increased availability of genetic testing, several aspects (absence of appropriate educational strategies, incomplete insurance coverage policies, inconsistent training for physicians, the particularity of each community in terms of psychosocial needs) still hinder access to genetic tests in some regions and populations [6]. The NEUROMYODredger‐3billion Megaproject was developed with the goal of providing free access to singleton whole exome sequencing (WES) for children and adults living with a genetically undiagnosed NDD or NMD in Algeria, Chile, Egypt, France, Mexico, Peru, and Romania. With the exception of France, free‐of‐charge genetic testing is either unavailable or difficult to obtain in the included low or middle‐income countries, and patients often need to seek costly alternatives [7]. Here we describe the obtained results while also presenting a collaborative approach as crucial for bridging existing gaps and creating a more inclusive framework for global patient support.

## 2 Materials and Methods

A total of 245 patients (including both children and adults) from 7 countries (Algeria, Chile, Egypt, France, Mexico, Peru, and Romania) were included in the project. The inclusion criteria were based on clinical and/or paraclinical evidence of neurodevelopmental or neuromuscular disorders. 168 patients (68.57%) were children and 77 (31.42%) were adults. Mean age was 9.3 ± 4.4 years in children and 45.4 ± 19.9 in adults. Overall, there were 140 male patients (57.14%) and 105 female patients (42.85%), respectively. Given the high clinical variability associated with these disorders, patients were classified into one of the following categories based upon the initial clinical suspicion: Neurodevelopmental delay (82, 33.47%), Muscular dystrophy (17, 6.94%), Limb‐girdle muscular dystrophy (15, 6.12%), Congenital myopathy (38, 15.51%), Metabolic myopathy (42, 17.14%), Distal myopathy (6, 2.45%), Myopathy (33, 13.47%), Neuropathy (2, 0.82%), Amyotrophic lateral sclerosis (4, 1.63%), Congenital myasthenic syndrome (4, 1.63%), and Skeletal dysplasia (2, 0.82%) (Tables S1 and S2).

The tested samples consisted of peripheral blood, dried blood spot (DBS) cards, or muscle tissue specimens. Singleton WES was performed by 3billion Inc., Republic of Korea, on a NovaSeq 6000 equipment (Illumina, San Diego, CA, USA), using the xGen Exome Research Panel v2, supplemented with the xGen human mtDNA panel and the xGen Custom Hyb Panel v1 (Integrated DNA Technologies, Coralville, Iowa, USA). Sequencing data analysis and variant interpretation were realized using 3billion's proprietary system, EVIDENCE v4.1, an automated variant prioritization system that has made the diagnostic process faster and more efficient [8].

This study was approved by the ethical committees of the different institutions involved and was performed according to the Declaration of Helsinki. Informed consent forms regarding participation in this project have been obtained from all patients or their legal guardians.

## 3 Results

**FIGURE 1:**
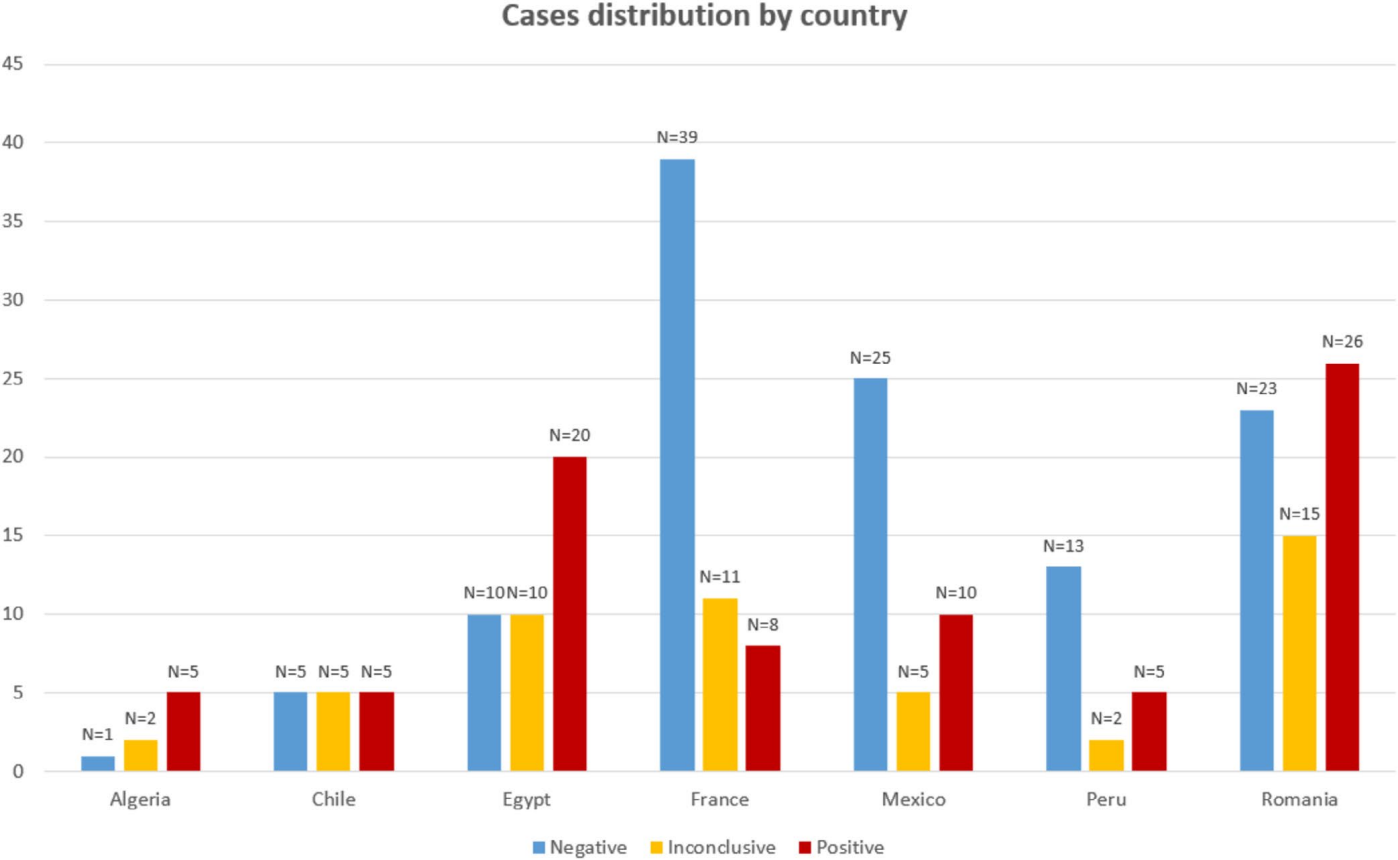
Cases distribution and diagnostic outcomes by country. This figure shows the number of positive, inconclusive, and negative cases from each country; the important number of negative cases in the French cohort could be explained by the fact that these patients have benefited from extensive investigations (including genetic tests) prior to their inclusion in this project.

Pathogenic variants were identified in 79 individuals, corresponding to a diagnostic rate of 32.24%. The diagnostic rate varied by country, ranging from 13.79% to 62.5%. This variation may be attributed to several factors, primarily differences in the number of cases included from each country (Figure 1) and the clinical heterogeneity of the cohort.

Additionally, 50 patients (20.41%) harbored variants of uncertain significance (VUS), which require further investigation. In the remaining 116 cases (47.35%), no pathogenic, likely pathogenic variants, or variants of uncertain significance were detected.

**FIGURE 2:**
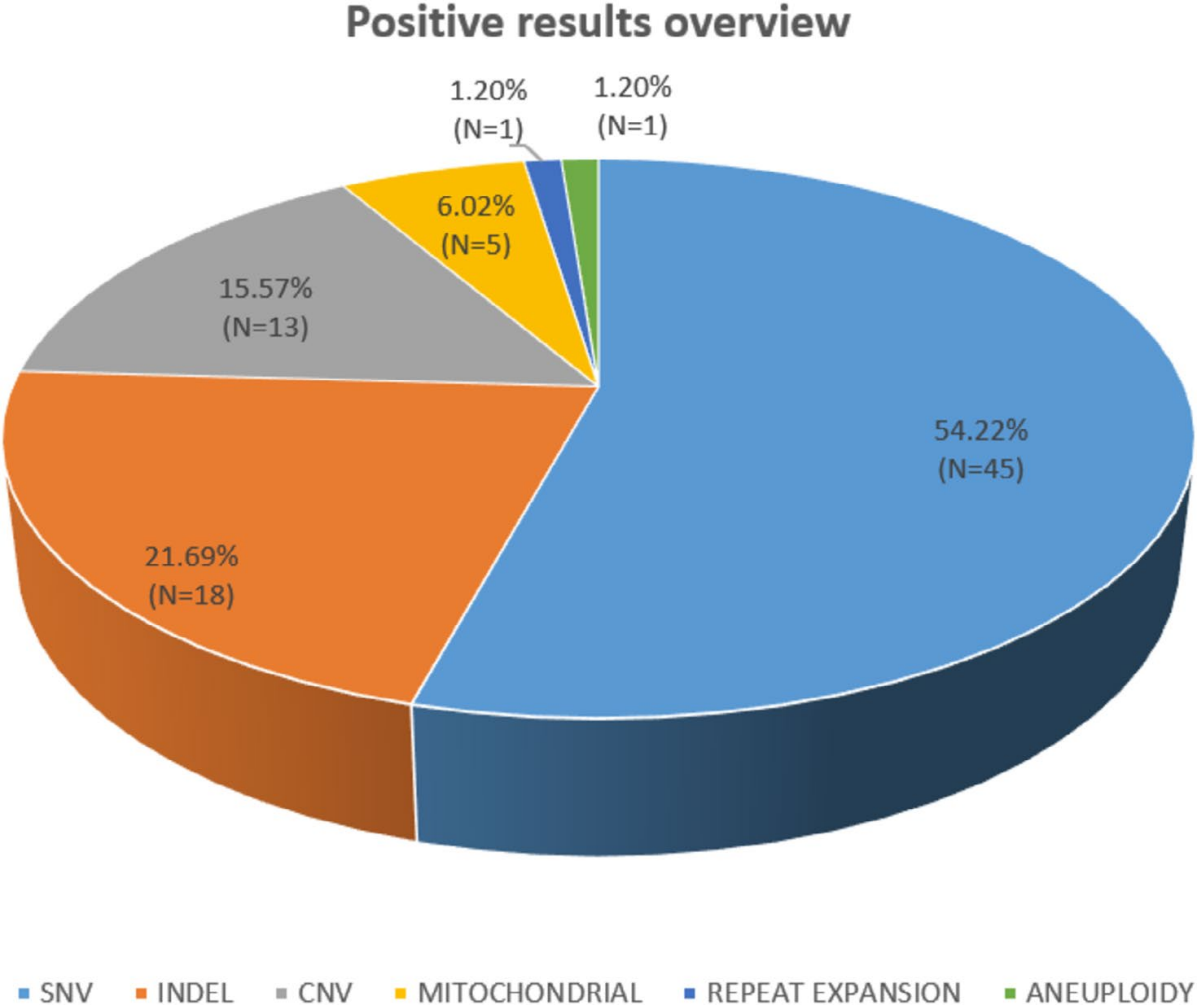
Positive results overview. There were 79 positive results from a total of 245 tested patients. There were 4 compound heterozygous patients and a total of 83 identified variants. The genetic anomalies identified were heterogeneous, with the most common types being single nucleotide variations and indels; there was also a significant number of copy number variations.

Among the positive cases, the most frequently identified variants were single nucleotide variants (SNVs) and small insertions/deletions (indels) (Figure 2). The complete list of identified variants is provided in Table S3.

## 4 Discussion

With the evolution of sequencing technology and the identification of large numbers of variants through this type of testing, an important challenge in the form of sequence data analysis has arisen. Currently, NGS data is analyzed according to the guidelines developed by the American College of Medical Genetics and Genomics, Association for Molecular Pathology, and College of American Pathologists; however, applying these standards on genetic results alone is not always enough to obtain a diagnosis, and variants might require more data (segregation, functional studies) in order to be classified as pathogenic or benign [9].

In this study, 82 patients presented with a neurodevelopmental disorder (the largest cohort in our group); the diagnostic yield for this category was 43.90%. Given the clinical heterogeneity of NDDs and the fact that most of our patients in this category are syndromic, we consider this diagnostic rate similar to other reported data in the literature—36% overall, 53% for neurodevelopmental disorders associated with other conditions [10].

Our cohort of neuromuscular patients included 163 patients belonging to several pathology subgroups and had a diagnostic yield of 26.38% (43 positive results). This diagnostic rate for singleton WES is smaller compared to other studies: 46% in a paediatric cohort [11], 63.4% in an adult cohort [12]; however, singleton WES had a 26% diagnostic rate in a group of 50 NMD paediatric patients [13], with various percentages for different NMD groups. These diagnostic rate variations could be explained by the geographical differences between cohorts, as well as the significant genetic and clinical variability of neuromuscular disorders, since these could be determined by various gene defects that could impact the nerves, nerve‐muscle junctions, or muscles, creating a wide symptomatology range [4, 14]. Also, we believe this heterogeneity is the main limitation of our project, as the results are hard to integrate in a general context because of it. In our cohort, another important aspect to mention is the lower diagnostic yield for the French patients (13.79%, 8 positive results from 58 patients); this is a detail we have expected, as France is a country with more resources compared to the others, and most patients from France had undergone several clinical, biological, myopathological, imaging, and genetic evaluations before the WES testing. Poor access to muscle MRI imaging and muscle biopsies in low‐ and middle‐income countries is another issue worth mentioning, as these investigations could have offered significant diagnosis‐orienting details.

The benefits of this project are similar, but slightly different in each of the involved countries. In Chile, obtaining access to exome testing or an NGS panel requires patients to pay for testing in most cases, without receiving any reimbursement. The situation is similar in Egypt, where genetic testing is still not covered by insurance. In Mexico, and probably in Latin America, clinical exome testing is not routinely performed in public institutions, and although more private laboratories are offering this study, the costs are not affordable for most patients. In Romania, WES is available for free only in two public genetics centres, and the turnaround time could go up to several months or more than 1 year because of a lack of funding, a reduced testing capacity, or socioeconomic issues such as low general awareness, distance, and poverty. Therefore, for the moment, this diagnostic tool is still not widely available in the included countries, making it even more difficult to reach a definitive diagnosis.

The collaboration that has been developed between the participating centres should be used for the further development of genetic testing strategies, helping in areas considered as key requirements for an equitable and timely access to genetic testing (testing service capacity, comprehensive clinical evaluation, interpretation accuracy, standardized reporting, multimodal approach, reanalysis strategy, patient feedback, and innovation through research and development) [15].

Moreover, raw sequencing data from the cases with inconclusive (VUS) and negative results are currently undergoing further analysis to identify potential novel variants that may have been overlooked by initial variant‐calling pipelines or were previously classified as non‐pathogenic due to a lack of sufficient evidence. Apart from re‐evaluating the existing data, further genomic investigations such as trio genome sequencing or RNASeq, which have proven to be effective [16], could be taken into account.

The use of singleton WES was determined by the requirements of the grant, as it was a specified condition of the funding. Additionally, our cohort data indicate that obtaining parental samples can be challenging in low‐ and middle‐income countries due to logistical and socioeconomic barriers, and the implementation of trio WES is often hindered by its higher cost, making it less feasible as a routine diagnostic approach. Given these limitations, we sought to demonstrate that singleton WES, when combined with CNV detection, mobile element insertion analysis, repeat expansion variant identification, and mitochondrial DNA analysis, is a powerful and effective tool for diagnosing these disorders.

## 5 Conclusion

WES remains an important diagnostic tool for neuromuscular patients, having an expected diagnostic yield of around 50%, and testing strategies which include WES and reanalysis protocols or complementary tests could offer better diagnosis management chances [17]. The NEUROMYODredger‐3billion Megaproject represents an initiative developed through a significant international collaboration to improve access to comprehensive genetic testing through WES for patients with NDDs or NMDs in several medically underserved regions. Beyond the diagnosed patients, this project also aims to add to existing genes and variants databases, contributing to the understanding of genetic disease mechanisms. The network of specialists that has been created will continue to work towards better genetic and medical services for the patients in the long term. A collaborative approach plays a key role in closing current gaps and establishing a more inclusive framework that provides support to patients globally.

## Author Contributions

Conceptualization: Homa Tajsharghi and Edoardo Malfatti. Data curation and formal analysis: Hussein Shoaito, Alexandru Caramizaru, and Andreea Dumitrescu. Funding acquisition: Homa Tajsharghi. Investigation: all authors. Methodology: Homa Tajsharghi and Edoardo Malfatti. Project administration: Edoardo Malfatti and Homa Tajsharghi. Resources: all authors. Software: Hane Lee and JiHye Kim. Supervision: Edoardo Malfatti and Homa Tajsharghi. Visualization: Edoardo Malfatti and Homa Tajsharghi. Writing – original draft preparation: Edoardo Malfatti and Alexandru Caramizaru. Writing – review and editing: Edoardo Malfatti, Hane Lee, JiHye Kim, Alessandra Pennisi, Sarah Souvannanorath, François‐Jérôme Authier, Nagia Fahmy, Rosa Elena Escobar‐Cedillo, Antonio Miranda‐Duarte, Sonia Nouioua, Ouissem Benchaabi, Meriem Tazir, Sihem Hallal, Peggy Martinez, Claudia Castiglioni, Amelia Dobrescu, and Homa Tajsharghi.

## Conflicts of Interest

The authors declare no conflicts of interest.

## Peer Review

The peer review history for this article is available at https://www.webofscience.com/api/gateway/wos/peer‐review/10.1111/cge.14736.

## Supporting information


**Supplementary Table 1** General findings according to origin in child participants. This table shows the number of positive, inconclusive and negative results for the child participants in the project (168) while also taking into consideration their gender and initial clinical suspicion.

**Supplementary Table 2** General findings according to origin in adult participants. This table shows the number of positive, inconclusive and negative results for the adult participants in the project (77) while also taking into consideration their gender and initial clinical suspicion.

**Supplementary Table 3** Variants identified in positive cases. This table contains a description of each variant identified in the positive cases. Patients with a clinical suspicion of spinal muscular atrophy, myotonic dystrophy type 1, or facioscapulohumeral dystrophy were excluded from the study, as some patients have been previously tested for these conditions. Patients clinically suspected for a dystrophinopathy were included if their clinical presentation was atypical or if alternative genetic testing options were unavailable at the time. Notably, a high number of homozygous variants (*n* = 27), and splicing‐altering variants (*n* = 9) were observed. Furthermore, 16 novel variants affecting 14 different genes were identified in this cohort. These genes are associated with well‐characterized disorders, as well as conditions with fewer than 100 reported cases, such as Arboleda‐Tham syndrome and ZTTK syndrome, where additional natural disease history data could be valuable. Interestingly, among the five patients with mitochondrial DNA anomalies, three had muscle DNA sample analysed, while the remaining two were assessed using a buccal swab and a dried blood spot (DBS) card sample, respectively. The test can detect single nucleotide variants (SNVs), small insertions/deletions (INDEL, < 50 bp), large copy number variants (≥ 3 consecutive exons), mobile element insertion variants and repeat expansion variants within the targeted genomic regions. Detection of repeat expansion variants is limited to 17 specific genes (*AR*, *ARX*, *ATN1*, *ATXN1*, *ATXN2*, *ATXN3*, *ATXN7*, *CACNA1A*, *COMP*, *FOXL2*, *HOXD13*, *HTT*, *PABPN1*, *PHOX2B*, *PRDM12*, *TBP*, and *ZIC2*). Additionally, within the mitochondrial genome, only SNV/INDEL variants with a heteroplasmic level greater than 10% are reported. Country abbreviations: DZA—Algeria; CHL—Chile; EGY—Egypt; FRA—France; MEX—Mexico; PER—Peru; ROU—Romania. Patients DZA04 and DZA05 are brothers.

## Data Availability

The patients' initial clinical suspicions are available in Tables S1 and S2. The positive results are available in Table S3 (available online).
