# Supplementary material for: NEUROMYODredger: Whole Exome Sequencing for the Diagnosis of Neurodevelopmental and Neuromuscular Disorders in Seven Countries
Source: Clin Genet. 2025 Feb 25;108(3):318–22. doi: 10.1111/cge.14736 (PMC12319130; doi:10.1111/cge.14736)
Supplement: Supplementary file 1 — Supplementary Table 1 General findings according to origin in child participants. This table shows the number of positive, inconclusive and negative results for the child participants in the project (168) while also taking into consideration their gender and initial clinical suspicion. [file CGE-108-318-s002.docx]

Table 1. General findings according to origin in child participants

| **Country of Origin** | **Gender** | **Initial suspicion** | **Result** | **Quantity** |
| --- | --- | --- | --- | --- |
| Algeria | Female | Congenital myopathy | Inconclusive | 1 |
|  | Male |  | Positive | 2 |
|  | Female |  | Positive | 3 |
|  | Female | Metabolic myopathy | Negative | 1 |
| Chile | Female | Distal myopathy | Negative | 1 |
|  | Male |  | Inconclusive | 1 |
|  | Male | Myopathy | Negative | 1 |
|  | Female | Neurodevelopmental delay | Negative | 2 |
|  | Male |  | Negative | 1 |
|  | Female |  | Inconclusive | 2 |
|  | Male |  | Inconclusive | 1 |
|  | Female |  | Positive | 2 |
|  | Male |  | Positive | 3 |
| Egypt | Male | LGMD | Positive | 2 |
|  | Female | Muscular dystrophy | Positive | 2 |
|  | Male |  | Positive | 1 |
|  | Female | Myopathy | Negative | 6 |
|  | Male |  | Negative | 2 |
|  | Male |  | Inconclusive | 2 |
|  | Female |  | Inconclusive | 2 |
|  | Male |  | Positive | 3 |
|  | Female |  | Positive | 2 |
|  | Male | Neurodevelopmental delay | Positive | 1 |
|  | Male | Neuropathy | Positive | 1 |
| France | Female | Congenital myopathy | Negative | 1 |
|  | Female | Metabolic myopathy | Negative | 1 |
|  | Male | Muscular dystrophy | Negative | 2 |
|  | Male | Neurodevelopmental delay | Negative | 1 |
| Mexico | Male | Congenital myopathy | Negative | 3 |
|  | Female |  | Negative | 1 |
|  | Male |  | Inconclusive | 4 |
|  | Male |  | Positive | 3 |
|  | Male | Distal myopathy | Negative | 2 |
|  | Female | Metabolic myopathy | Negative | 4 |
|  | Male |  | Negative | 5 |
|  | Female |  | Positive | 1 |
|  | Male | Muscular dystrophy | Positive | 3 |
|  | Female |  | Positive | 1 |
|  | Female | Neurodevelopmental delay | Negative | 1 |
|  | Male |  | Negative | 7 |
|  | Male |  | Inconclusive | 1 |
|  | Male |  | Positive | 2 |
| Peru | Male | Congenital myasthenic syndrome | Negative | 2 |
|  | Female |  | Inconclusive | 1 |
|  | Female | Congenital myopathy | Negative | 1 |
|  | Male |  | Negative | 4 |
|  | Male |  | Inconclusive | 1 |
|  | Female | Distal myopathy | Negative | 1 |
|  | Male | LGMD | Negative | 1 |
|  | Male | Metabolic myopathy | Negative | 2 |
|  | Female |  | Positive | 1 |
|  | Male | Muscular dystrophy | Negative | 1 |
|  | Female |  | Negative | 1 |
|  | Male | Myopathy | Positive | 1 |
|  | Female | Neurodevelopmental delay | Negative | 1 |
|  | Male |  | Positive | 1 |
|  | Female | Skeletal dysplasia | Positive | 1 |
| Romania | Female | LGMD | Inconclusive | 1 |
|  | Female | Metabolic myopathy | Negative | 1 |
|  | Male | Muscular dystrophy | Inconclusive | 3 |
|  | Female | Myopathy | Negative | 1 |
|  | Male |  | Inconclusive | 2 |
|  | Male | Neurodevelopmental delay | Negative | 10 |
|  | Female |  | Negative | 8 |
|  | Male |  | Inconclusive | 6 |
|  | Female |  | Inconclusive | 3 |
|  | Male |  | Positive | 13 |
|  | Female |  | Positive | 12 |
|  |  |  | **Total** | **168** |
