# Supplementary material for: NEUROMYODredger: Whole Exome Sequencing for the Diagnosis of Neurodevelopmental and Neuromuscular Disorders in Seven Countries
Source: Clin Genet. 2025 Feb 25;108(3):318–22. doi: 10.1111/cge.14736 (PMC12319130; doi:10.1111/cge.14736)
Supplement: Supplementary file 2 — Supplementary Table 2 General findings according to origin in adult participants. This table shows the number of positive, inconclusive and negative results for the adult participants in the project (77) while also taking into consideration their gender and initial clinical suspicion. [file CGE-108-318-s001.docx]

Table 2. General findings according to origin in adult participants.

| **Country of Origin** | **Gender** | **Initial suspicion** | **Result** | **Quantity** |
| --- | --- | --- | --- | --- |
| Algeria | Female | Congenital myopathy | Inconclusive | 1 |
| Chile | Female | Distal myopathy | Inconclusive | 1 |
| Egypt | Male | Congenital myasthenic syndrome | Inconclusive | 1 |
|  | Male | LGMD | Inconclusive | 1 |
|  | Female |  | Inconclusive | 1 |
|  | Female |  | Positive | 4 |
|  | Male |  | Positive | 2 |
|  | Male | Myopathy | Negative | 1 |
|  | Female |  | Negative | 1 |
|  | Male |  | Inconclusive | 1 |
|  | Female |  | Inconclusive | 1 |
|  | Male |  | Positive | 2 |
|  | Female | Neurodevelopmental delay | Inconclusive | 1 |
| France | Female | Amyotrophic lateral sclerosis | Negative | 2 |
|  | Male |  | Inconclusive | 1 |
|  | Male |  | Positive | 1 |
|  | Female | Congenital myopathy | Negative | 4 |
|  | Male |  | Negative | 5 |
|  | Female |  | Inconclusive | 2 |
|  | Male |  | Inconclusive | 1 |
|  | Male |  | Positive | 1 |
|  | Male | LGMD | Negative | 2 |
|  | Female | Metabolic myopathy | Negative | 11 |
|  | Male |  | Negative | 3 |
|  | Female |  | Inconclusive | 2 |
|  | Male |  | Inconclusive | 4 |
|  | Male |  | Positive | 2 |
|  | Female |  | Positive | 3 |
|  | Male | Muscular dystrophy | Negative | 2 |
|  | Female | Myopathy | Negative | 2 |
|  | Male |  | Negative | 2 |
|  | Male |  | Inconclusive | 1 |
|  | Female | Neurodevelopmental delay | Positive | 1 |
|  | Male | Neuropathy | Negative | 1 |
| Mexico | Male | Metabolic myopathy | Negative | 1 |
|  | Male | Muscular dystrophy | Negative | 1 |
| Romania | Male | LGMD | Negative | 1 |
|  | Male | Neurodevelopmental delay | Negative | 1 |
|  | Female |  | Positive | 1 |
|  | Male | Skeletal dysplasia | Negative | 1 |
|  |  |  | **Total** | **77** |
