# Supplementary material for: NEUROMYODredger: Whole Exome Sequencing for the Diagnosis of Neurodevelopmental and Neuromuscular Disorders in Seven Countries
Source: Clin Genet. 2025 Feb 25;108(3):318–22. doi: 10.1111/cge.14736 (PMC12319130; doi:10.1111/cge.14736)
Supplement: Supplementary file 3 — Supplementary Table 3 Variants identified in positive cases. This table contains a description of each variant identified in the positive cases. Patients with a clinical suspicion of spinal muscular atrophy, myotonic dystrophy type 1, or facioscapulohumeral dystrophy were excluded from the study, as some patients have been previously tested for these conditions. Patients clinically suspected for a dystrophinopathy were included if their clinical presentation was atypical or if alternative genetic testing options were unavailable at the time. Notably, a high number of homozygous variants (n = 27), and splicing‐altering variants (n = 9) were observed. Furthermore, 16 novel variants affecting 14 different genes were identified in this cohort. These genes are associated with well‐characterized disorders, as well as conditions with fewer than 100 reported cases, such as Arboleda‐Tham syndrome and ZTTK syndrome, where additional natural disease history data could be valuable. Interestingly, among the five patients with mitochondrial DNA anomalies, three had muscle DNA sample analysed, while the remaining two were assessed using a buccal swab and a dried blood spot (DBS) card sample, respectively. The test can detect single nucleotide variants (SNVs), small insertions/deletions (INDEL, < 50 bp), large copy number variants (≥ 3 consecutive exons), mobile element insertion variants and repeat expansion variants within the targeted genomic regions. Detection of repeat expansion variants is limited to 17 specific genes (AR, ARX, ATN1, ATXN1, ATXN2, ATXN3, ATXN7, CACNA1A, COMP, FOXL2, HOXD13, HTT, PABPN1, PHOX2B, PRDM12, TBP, and ZIC2). Additionally, within the mitochondrial genome, only SNV/INDEL variants with a heteroplasmic level greater than 10% are reported. Country abbreviations: DZA—Algeria; CHL—Chile; EGY—Egypt; FRA—France; MEX—Mexico; PER—Peru; ROU—Romania. Patients DZA04 and DZA05 are brothers. [file CGE-108-318-s003.docx]

Table 3. Variants identified in positive cases

| Patient | DOB | Gene | Variant | Details | Disease |
| --- | --- | --- | --- | --- | --- |
| DZA04 | 18/12/2018 | *TNNT1* | Genomic Position: 19-55648576-TCTGC-T (GRCh37) DNA: NM_003283.6:c.502_505del Protein: NP_003274.3:p.(Ala168Asn)fsTer13 Zygosity: Homozygous  Pathogenic | New variant | Nemaline myopathy 5B, autosomal recessive, childhood-onset (OMIM: 620386), autosomal recessive |
| DZA05 | 22/05/2022 | *TNNT1* | Genomic Position: 19-55648576-TCTGC-T (GRCh37) DNA: NM_003283.6:c.502_505del Protein: NP_003274.3:p.(Ala168Asn)fsTer13 Zygosity: Homozygous  Pathogenic | New variant | Nemaline myopathy 5B, autosomal recessive, childhood-onset (OMIM: 620386), autosomal recessive |
| DZA06 | 17/06/2016 | *ACTA1* | Genomic Position: 1-229568620-A-G (GRCh37) DNA: NM_001100.4:c.137T>C Protein: NP_001091.1:p.(Met46Thr) Zygosity: Heterozygous  Likely Pathogenic | Known variant [1] | Myopathy, scapulohumeroperoneal (OMIM: 616852), autosomal dominant |
| DZA07 | 28/03/2016 | *RYR1* | Genomic Position: 19-39071079-C-T (GRCh37) DNA: NM_000540.3:c.14581C>T Protein: NP_000531.2:p.(Arg4861Cys) Zygosity: Heterozygous  Likely Pathogenic | Known variant [2] | Congenital myopathy 1A, autosomal dominant, with susceptibility to malignant hyperthermia (OMIM: 117000), autosomal dominant |
| DZA08 | 24/12/2007 | *RYR1* | Genomic Position: 19-39075614-G-A (GRCh37) DNA: NM_000540.3:c.14678G>A Protein: NP_000531.2:p.(Arg4893Gln) Zygosity: Heterozygous  Likely Pathogenic | Known variant [2] | Congenital myopathy 1A, autosomal dominant, with susceptibility to malignant hyperthermia (OMIM: 117000), autosomal dominant |
| CHL11 | 19/05/2009 | *TTN* | Genomic Position: 2-179518195-GTTTTC-G (GRCh37) DNA: NM_001267550.2:c.38661_38665del Protein: NP_001254479.2:p.(Lys12887Asn)fsTer6 Zygosity: Homozygous  Pathogenic | Known variant [3] | *TTN*-related  myopathy (OMIM: 188840), autosomal recessive |
| CHL12 | 04/02/2019 | *EXOSC3* | Genomic Position: 9-37783990-T-G (GRCh37) DNA: NM_016042.4:c.395A>C Protein: NP_057126.2:p.(Asp132Ala) Zygosity: Homozygous  Pathogenic | Known variant [4, 5] | Pontocerebellar hypoplasia, type 1B (OMIM: 614678), autosomal recessive |
| CHL13 | 14/01/2015 | *SOX2* | Genomic Position: 3-181430201-C-CG (GRCh37) DNA: NM_003106.4:c.59dup Protein: NP_003097.1:p.(Gly21Arg)fsTer75 Zygosity: Heterozygous  Pathogenic | Known variant [6] | Optic nerve hypoplasia and abnormalities of the central nervous system (OMIM: 206900), autosomal dominant |
| CHL14 | 11/03/2011 | *SPTBN4* | Genomic Position: 19-41035039-G-A (GRCh37) DNA: NM_020971.3:c.3948+1G>A Protein: NP_066022.2:p.? Zygosity: Heterozygous  Likely Pathogenic | New variant | Neurodevelopmental disorder with hypotonia, neuropathy, and deafness (OMIM: 617519), autosomal recessive |
|  |  |  | Genomic Position: 19-41074058-G-T (GRCh37 DNA: NM_020971.3:c.6826G>T Protein: NP_066022.2:p.(Glu2276Ter) Zygosity: Heterozygous  Likely Pathogenic | New variant |  |
| CHL15 | 28/04/2011 | *SPTAN1* | Genomic Position: 9-131356530-C-T (GRCh37) DNA: NM_001130438.3:c.3292C>T Protein: NP_001123910.1:p.(Arg1098Cys) Zygosity: Heterozygous  Likely Pathogenic | Known variant [7] | Developmental and epileptic encephalopathy 5 (OMIM: 613477), autosomal recessive |
| EGY21 | 10/06/2008 | *POMT2* | Genomic Position: 14-77769283-G-A (GRCh37) DNA: NM_013382.7:c.551C>T Protein: NP_037514.2:p.(Thr184Met) Zygosity: Homozygous  Likely Pathogenic | Known variant [8] | *POMT2*-related disorder  (OMIM: 607439), autosomal recessive |
| EGY22 | 14/07/2017 | *LMNA* | Genomic Position: 1-156084825-A-G (GRCh37) DNA: NM_170707.4:c.116A>G Protein: NP_733821.1p.(Asn39Ser) Zygosity: Heterozygous  Pathogenic | Known variant [9, 10] | *LMNA*-related disorder (OMIM: 150330), autosomal dominant |
| EGY23 | 01/01/2006 | *SGCG* | Genomic Position: 13-23777877-T-TAG (GRCh37) DNA: NM_000231.3:c.49_50dup Protein: NP_000222.2:p.(Pro18Gly)fsTer33 Zygosity: Homozygous  Pathogenic | New variant | Muscular dystrophy, limb-girdle, autosomal recessive 5 (OMIM: 253700), autosomal recessive |
| EGY24 | 01/01/2013 | *MFN2* | Genomic Position: 1-12052716-C-T (GRCh37) DNA: NM_014874.4:c.280C>T Protein: NP_055689.1:p.(Arg94Trp) Zygosity: Heterozygous  Pathogenic | Known variant [11-15] | Charcot-Marie-Tooth disease, axonal, type 2A2A (OMIM: 609260), autosomal dominant |
| EGY25 | 01/01/2008 | *GNE* | Genomic Position: 9-36217396-A-G (GRCh37) DNA: NM_005476.7:c.2135T>C Protein: NP_005467.1:p.(Met712Thr) Zygosity: Homozygous  Pathogenic | Known variant [16] | Nonaka myopathy (OMIM: 605820), autosomal recessive |
| EGY26 | 01/01/1996 | *CAPN3* | Genomic Position: 15-42702183-C-T (GRCh37) DNA: NM_000070.3:c.2105C>T Protein: NP_000061.1:p.(Ala702Val) Zygosity: Homozygous  Likely Pathogenic | Known variant [17-19] | Muscular dystrophy, limb-girdle, autosomal recessive 1 (OMIM: 253600), autosomal recessive |
| EGY27 | 01/01/1980 | *PYROXD1* | Genomic Position: 12-21605064-A-G (GRCh37) DNA: NM_024854.5:c.464A>G Protein: NP_079130.2:p.(Asn155Ser) Zygosity: Homozygous  Pathogenic | Known variant [20] | Myopathy, myofibrillar, 8 (OMIM: 617258), autosomal recessive |
| EGY28 | 01/01/2014 | *COL6A1* | Genomic Position: 21-47410741-G-A (GRCh37) DNA: NM_001848.3:c.1056+1G>A Protein: NP_001839.2:p.? Zygosity: Heterozygous  Pathogenic | Known variant [21] | Bethlem myopathy 1 (OMIM: 158810), autosomal dominant |
| EGY29 | 01/01/2013 | *FKRP* | Genomic Position: 19-47259605-G-A (GRCh37) DNA: NM_024301.5:c.898G>A Protein: NP_077277.1:p.(Val300Met) Zygosity: Homozygous  Pathogenic | Known variant [22] | Muscular dystrophy-dystroglycanopathy (limb-girdle), type C, 5 (OMIM: 607155), autosomal recessive |
| EGY30 | 17/01/2002 | *SGCA* | Genomic Position: 17-48245923-C-T (GRCh37) DNA: NM_000023.4:c.574C>T Protein: NP_000014.1:p.(Arg192Ter) Zygosity: Homozygous  Pathogenic | Known variant [23, 24] | Muscular dystrophy, limb-girdle, autosomal recessive 3 (OMIM: 608099), autosomal recessive |
| EGY31 | 02/03/2001 | *FKRP* | Genomic Position: 19-47259252-A-G (GRCh37) DNA: NM_024301.5:c.545A>G Protein: NP_077277.1:p.(Tyr182Cys) Zygosity: Homozygous  Pathogenic | Known variant [22] | *FKRP*-related disorder (OMIM: 606596), autosomal recessive |
| EGY32 | 01/01/2010 | *SPTAN1* | Genomic Position: 9-131365842-C-CA (GRCh37) DNA: NM_001130438.3:c.3601dup Protein: NP_001123910.1:p.(Thr1201Asn)fsTer6 Zygosity: Heterozygous  Likely Pathogenic | New variant | Developmental and epileptic encephalopathy 5 (OMIM: 613477), autosomal dominant |
| EGY33 | 06/12/2006 | *LMNA* | Genomic Position: 1-156106204-C-T (GRCh37) DNA: NM_170707.4:c.1357C>T Protein: NP_733821.1:p.(Arg453Trp) Zygosity: Heterozygous  Pathogenic | Known variant [25-28] | *LMNA*-related disorder  (OMIM: 150330), autosomal dominant |
| EGY34 | 25/03/1991 | *SGCG* | Genomic Position: 13-23869568-CT-C (GRCh37) DNA: NM_000231.3:c.525del Protein: NP_000222.2:p.(Phe175Leu)fsTer20 Zygosity: Homozygous  Pathogenic | Known variant [29] | Muscular dystrophy, limb-girdle, autosomal recessive 5 (OMIM: 253700), autosomal recessive |
| EGY35 | 13/10/2002 | *CAPN3* | Genomic Position: 15-42680000-CA-C (GRCh37) DNA: NM_000070.3:c.550del Protein: NP_000061.1:p.(Thr184Arg)fsTer36 Zygosity: Homozygous  Pathogenic | Known variant [30] | Muscular dystrophy, limb-girdle, autosomal recessive 1 (OMIM: 253600), autosomal recessive |
| EGY36 | 03/02/2002 | *CLCN1* | Genomic Position: 7-143036416-G-A (GRCh37) DNA: NM_000083.3:c.1471+1G>A Protein: NP_000074.3:p.? Zygosity: Heterozygous  Pathogenic | Known variant [31] | Myotonia congenita, recessive (OMIM: 255700), autosomal recessive |
|  |  |  | Genomic Position: 7-143048876-AC-A (GRCh37) DNA: NM_00008.3:c.2789del Protein: NP_000074.3:p.(Pro930Leu)fsTer18 Zygosity: Heterozygous  Likely Pathogenic | Known variant [32] |  |
| EGY37 | 23/06/2011 | *CLCN1* | Genomic Position: 7-143036388-G-A (GRCh37) DNA: NM_000083.3:c.1444G>A Protein: NP_000074.3:p.(Gly482Arg) Zygosity: Homozygous  Pathogenic | Known variant [31] | Myotonia congenita, recessive (OMIM: 255700), autosomal recessive |
| EGY38 | 01/01/2012 | *COL6A2* | Genomic Position: 21-47546037-G-GA (GRCh37) DNA: NM_001849.4:c.2312dup Protein: NP_001840.3:p.(Asn771Lys)fsTer25 Zygosity: Homozygous  Pathogenic | Known variant [33] | *COL6A2* -related disorder (OMIM: 120240), autosomal recessive |
| EGY39 | 02/12/2013 | *TTN* | Genomic Position: 2-179436521-G-A (GRCh37) DNA: NM_001267550.2:c.74338C>T Protein: NP_001254479.2:p.(Arg24780Ter) Zygosity: Heterozygous  Pathogenic | Known variant [34] | Salih myopathy (OMIM: 611705), autosomal recessive |
|  |  |  | Genomic Position: 2-179531574-CT-C (GRCh37) DNA: NM_001267550.2:c.35851del Protein: NP_001254479.2:p.(Arg11951Glu)fsTer19 Zygosity: Heterozygous  Likely Pathogenic | New variant |  |
| EGY40 | 25/11/2013 | *GMPPB* | Genomic Position: 3-49760132-G-A (GRCh37) DNA: NM_021971.4:c.458C>T Protein: NP_068806.2:p.(Thr153Ile) Zygosity: Homozygous  Pathogenic | Known variant [35-38] | *GMPPB*-related disorder (OMIM:615320), autosomal recessive |
| FRA51 | 09/08/1990 | *PLCXD1, GTPBP6 +44 more genes* | Genomic Position: NC_000023.10:g.(?_200854)_(12628000_?)del (GRCh37)  Cytogenetic band: Xp22.33p22.2 (minimum size: 12.4Mb) Type: Deletion Zygosity: Heterozygous  Pathogenic | Known region | Autosomal  dominant Xp22.3 microdeletion syndrome (ORPHA: 1643), autosomal dominant |
| FRA52 | 10/12/1992 | *MYH3* | Genomic Position: 17-10559406-C-T (GRCh37) DNA: NM_002470.4:c.-9+1G>A Protein: NP_002461.2:p.? Zygosity: Homozygous  Pathogenic | Known variant [39] | Contractures, pterygia, and spondylocarpotarsal fusion syndrome 1B (OMIM: 618469), autosomal recessive |
| FRA53 | 28/02/1967 | *PNPLA2* | Genomic Position: 11-824119-CT-C (GRCh37) DNA: NM_020376.4:c.1043del Protein: NP_065109.1:p.(Phe348Ser)fsTer19 Zygosity: Homozygous  Pathogenic | Known variant [40] | Neutral lipid storage disease with myopathy (OMIM: 610717), autosomal recessive |
| FRA54 | 04/03/1950 | *TPM2* | Genomic Position: 9-35689792-ATCT-A (GRCh37) DNA: NM_003289.4:c.20_22del Protein: NP_003280.2:p.(Lys7del) Zygosity: Heterozygous  Pathogenic | Known variant [41-43] | Congenital myopathy 23 (OMIM: 609285), autosomal dominant |
| FRA55 | 28/07/1962 | *DMD* | Genomic Position: NC_000023.10:g.(?_32715987)_(32867937_?)del (GRCh37)  Cytogenetic band: Xp21.1 (minimum size: 151.9Kb)  Type: Deletion  Zygosity: Hemizygous  Pathogenic | Known variant [44] | Becker muscular dystrophy (OMIM: 300376), X-linked recessive |
| FRA56 | 20/08/1942 | *MT-TRND, MT-COX2 +13 more genes* | Genomic Position: NC_012920.1:m.7561_12763del (GRCh37)   Cytogenetic band: MT (minimum size: 5.2Kb)  Type: Deletion  Zygosity: Heteroplasmic (VAF:~53%)  Pathogenic | Known region [45] | Kearns-Sayre syndrome (OMIM: 530000), mitochondrial |
| FRA57 | 25/05/1956 | *MT-ATP8, MT-ATP6 +10 more genes* | Genomic Position: NC_012920.1:m.8470_13446del (GRCh37)   Cytogenetic band: MT (minimum size: 5.0Kb)  Type: Deletion  Zygosity: Heteroplasmic (VAF:~69%)  Pathogenic | Known region [45] | Kearns-Sayre syndrome (OMIM: 530000), mitochondrial |
| FRA58 | 03/12/1949 | *MT-TL1* | Genomic Position: MT-3243-A-G (GRCh37)  DNA: NC_012920.1:m.3243A>G  Protein: Not applicable  Zygosity: Heteroplasmic (VAF:~68%)  Pathogenic | Known variant [46-51] | Mitochondrial myopathy, encephalopathy, lactic acidosis, and stroke-like episodes; MELAS (OMIM: 540000), mitochondrial |
| MEX31 | 21/10/2012 | *MT-TL1* | Genomic Position: MT-3243-A-G (GRCh37) DNA: NC_012920.1:m.3243A>G Protein: Not applicable Zygosity: Heteroplasmic (VAF:~19%)  Pathogenic | Known variant [46-54] | Mitochondrial myopathy, encephalopathy, lactic acidosis, and stroke-like episodes;  MELAS (OMIM: 540000), mitochondrial |
| MEX32 | 06/05/2011 | *ENTPD1* | Genomic Position: 10-97604355-G-A (GRCh37) DNA: NM_001776.6:c.536G>A Protein: NP_001767.3:p.(Trp179Ter) Zygosity: Homozygous  Pathogenic | Known variant [55] | Spastic paraplegia 64, autosomal recessive (OMIM: 615683), autosomal recessive |
| MEX33 | 21/05/2014 | *MTM1* | Genomic Position: X-149826515-C-G (GRCh37) DNA: NM_000252.3:c.1260+15C>G Protein: NP_000243.1:p.? Zygosity: Hemizygous  Likely Pathogenic | Known variant [56] | Myopathy, centronuclear, X-linked (OMIM: 310400), X-linked recessive |
| MEX34 | 21/06/2010 | *RYR1* | Genomic Position: 19-38986890-C-T (GRCh37) DNA: NM_000540.3:c.6584C>T Protein: NP_000531.2:p.(Pro2195Leu) Zygosity: Heterozygous  Likely Pathogenic | Known variant [57] | Congenital myopathy 1A, autosomal dominant, with susceptibility to malignant  hyperthermia (OMIM: 117000), autosomal dominant |
| MEX35 | 13/03/2009 | *PTEN* | Genomic Position: 10-89624266-A-G (GRCh37) DNA: NM_000314.8:c.40A>G Protein: NP_000305.3:p.(Arg14Gly) Zygosity: Heterozygous  Likely Pathogenic | Known variant [58] | Cowden syndrome 1 (OMIM: 158350), autosomal dominant |
| MEX36 | 10/12/2016 | *FKRP* | Genomic Position: 19-47260094-A-G (GRCh37) DNA: NM_024301.5:c.1387A>G Protein: NP_077277.1:p.(Asn463Asp) Zygosity: Homozygous  Pathogenic | Known variant [59] | Muscular dystrophy-dystroglycanopathy (congenital with or without impaired  intellectual development), type B, 5 (OMIM: 606612), autosomal recessive |
| MEX37 | 20/02/2012 | *DYNC1H1* | Genomic Position: 14-102452355-G-T (GRCh37)   DNA: NM_001376.5:c.1793G>T  Protein: NP_001367.2:p.(Arg598Leu)  Zygosity: Heterozygous  Pathogenic | Known variant [60] | Spinal muscular atrophy, lower extremity-predominant 1, AD (OMIM: 158600), autosomal dominant |
| MEX38 | 27/10/2018 | *DOK7* | Genomic Position: 4-3478218-G-A (GRCh37) DNA: NM_173660.5:c.481G>A Protein: NP_775931.3:p.(Gly161Arg) Zygosity: Heterozygous  Likely Pathogenic | Known variant [61] | Myasthenic syndrome, congenital, 10 (OMIM: 254300), autosomal recessive |
|  |  |  | Genomic Position: 4-3494969-GC-G (GRCh37) DNA: NM_173660.5:c.1263del Protein: NP_775931.3:p.(Ser422His)fsTer34 Zygosity: Heterozygous  Pathogenic | Known variant [62, 63] |  |
| MEX39 | 30/09/2014 | *DMD* | Genomic Position: NC_000023.10:g.(?_31986456)_(32827728_?)del (GRCh37)   Cytogenetic band: Xp21.1 (minimum size: 841.3Kb)  Type: Deletion  Zygosity: Heterozygous  Pathogenic | New variant | Duchenne muscular dystrophy (OMIM: 310200), X-linked recessive |
| MEX40 | 04/03/2011 | *LMNA* | Genomic Position: 1-156107458-G-A (GRCh37)  DNA: NM_170707.4:c.1622G>A  Protein: NP_733821.1:p.(Arg541His)  Zygosity: Heterozygous  Pathogenic | Known variant [64, 65] | Emery-Dreifuss muscular dystrophy 2, autosomal dominant (OMIM: 181350), autosomal dominant |
| PER16 | 16/06/2012 | *MT-TS1* | Genomic Position: MT-7465-A-AC (GRCh37) DNA: NC_012920.1:m.7471dup Protein: Not applicable Zygosity: Heteroplasmic (VAF:~20%)  Pathogenic | Known variant [66-71] | Mitochondrial myopathy, encephalopathy, lactic acidosis, and stroke-like episodes;  MELAS (OMIM: 540000), mitochondrial |
| PER17 | 08/08/2014 | *COMP* | Genomic Position: 19-18896844-T-TGTC (GRCh37) DNA: NM_000095.3:c.1405GAC[6] Protein: NP_000086.2:p.(Asp473dup) Zygosity: Heterozygous  Likely Pathogenic | Known variant [72] | Epiphyseal dysplasia, multiple, 1 (OMIM: 132400), autosomal dominant |
| PER18 | 10/09/2019 | *COL1A2* | Genomic Position: 7-94057661-T-C (GRCh37) DNA: NM_000089.4:c.3583T>C Protein: NP_000080.2:p.(Cys1195Arg) Zygosity: Heterozygous  Likely Pathogenic | Known variant [73] | Ehlers-Danlos syndrome, arthrochalasia type, 2 (OMIM: 617821), autosomal dominant |
| PER19 | 25/10/2012 | *COX10, CDRT15 +8 more genes* | Genomic Position: NC_000017.10:g.(?_14095306)_(15472344_?)del (GRCh37) Cytogenetic band: 17p12 (minimum size: 1.3Mb) Type: Deletion Zygosity: Heterozygous  Pathogenic | Known region [74] | Neuropathy, recurrent, with pressure palsies (OMIM: 162500), autosomal dominant |
| PER20 | 04/01/2009 | *ATL1* | Genomic Position: 14-51094854-G-A (GRCh37) DNA: NM_015915.5:c.1225G>A Protein: NP_056999.2:p.(Gly409Ser) Zygosity: Heterozygous  Likely Pathogenic | Known variant [75] | Spastic paraplegia 3A, autosomal dominant (OMIM: 182600), autosomal dominant |
| ROU39 | 17/07/2023 | *BPTF* | Genomic Position: 17-65887948-TTCATTTGACAGTAGG-C (GRCh37)   DNA: NM_182641.4:c.1865-12_1868delinsC  Protein: NP_872579.2:p.?  Zygosity: Heterozygous  Likely Pathogenic | New variant | Neurodevelopmental disorder with dysmorphic facies and distal limb anomalies (OMIM: 617755), autosomal dominant |
| ROU40 | 04/11/2017 | *FAM53B, EEF1AKMT2 +35 more genes* | Genomic Position: NC_000010.10:g.(?_126370176)_(134674486_?)del (GRCh37)  Cytogenetic band: 10q26.13q26.3 (minimum size: 8.3Mb)  Type: Deletion  Zygosity: Heterozygous  Pathogenic | Known region [76, 77] | Chromosome 10q26 deletion syndrome (OMIM: 609625), autosomal dominant |
| ROU41 | 10/01/2021 | *SCN8A* | Genomic Position: 12-52099295-T-C (GRCh37)  DNA: NM_001330260.2:c.1229T>C  Protein: NP_001317189.1:p.(Val410Ala)  Zygosity: Heterozygous  Likely Pathogenic | Known variant [78] | SCN8A-related disorder (OMIM: 600702), autosomal dominant |
| ROU42 | 12/10/2020 | *NR4A2, GPD2 +29 more genes* | Genomic Position: NC_000002.11:g.(?_157182256)_(163393590_?)del (GRCh37)  Cytogenetic band: 2q24.1q24.2 (minimum size: 6.21Mb)  Type: Deletion  Zygosity: Heterozygous  Pathogenic | Known region [79] | 2q24.1q24.2 deletion syndrome, autosomal dominant |
| ROU43 | 22/07/2014 | *47, XY +8* | Trisomy 8  Type: Gain  Pathogenic | Known aneuploidy | Trisomy 8 (ORPHA:96061), chromosomal |
| ROU44 | 21/04/2021 | *TBL1XR1, KCNMB2 +144 more genes* | Genomic Position: NC_000003.11:g.(?_176743286)_(197765538_?)dup (GRCh37)  Cytogenetic band: 3q26.32q29 (minimum size: 21Mb)  Type: Duplication  Pathogenic | Known region [80] | 3q26.32q29 duplication syndrome, autosomal dominant |
| ROU45 | 27/02/2016 | *SPN, QPRT +26 more genes* | Genomic Position: NC_000016.9:g.(?_29675050)_(30199897_?)del (GRCh37)  Cytogenetic band: 16p11.2 (minimum size: 524.8Kb)  Type: Deletion  Zygosity: Heterozygous  Pathogenic | Known region [81-83] | Chromosome 16p11.2 deletion syndrome, 593kb (OMIM: 611913), autosomal dominant |
| ROU46 | 25/03/2022 | *ZEB2* | Genomic Position: NC_000002.11:g.(?_145147018)_(145182434_?)del (GRCh37)  Cytogenetic band: 2q22.3 (minimum size: 35.4Kb)  Type: Deletion  Zygosity: Heterozygous  Pathogenic | New variant | Mowat-Wilson syndrome (OMIM: 235730), autosomal dominant |
| ROU47 | 18/11/2017 | *BBS7* | Genomic Position: 4-122775861-CCTCT-C (GRCh37) DNA: NM_176824.3:c.712_715del Protein: NP_789794.1:p.(Arg238Glu)fsTer59 Zygosity: Homozygous  Pathogenic | Known variant [84] | Bardet-Biedl syndrome 7 (OMIM: 615984), autosomal recessive |
| ROU48 | 13/02/2017 | *KAT6A* | Genomic Position: 8-41804108-C-G (GRCh37)  DNA: NM_006766.5:c.1996+1G>C  Protein: NP_006757.2:p.?  Zygosity: Heterozygous  Likely Pathogenic | New variant | Arboleda-Tham syndrome (OMIM: 616268), autosomal dominant |
| ROU49 | 09/07/2022 | *GALT* | Genomic Position: 9-34649029-G-T (GRCh37)  DNA: NM_000155.4:c.855G>T  Protein: NP_000146.2:p.(Lys285Asn)  Zygosity: Homozygous  Pathogenic | Known variant [85, 86] | Galactosemia (OMIM: 230400), autosomal recessive |
| ROU50 | 04/09/2010 | *DMD* | Genomic Position: NC_000023.10:g.(?_32429869)_(32717410_?)del (GRCh37)  Cytogenetic band: Xp21.1 (minimum size: 287.5Kb)  Type: Deletion  Zygosity: Hemizygous  Pathogenic | New variant | Duchenne muscular dystrophy (OMIM: 310200), X-linked recessive |
| ROU51 | 29/10/2012 | *TRAPPC11* | Genomic Position: 4-184605212-G-A (GRCh37)  DNA: NM_021942.6:c.1287+5G>A  Protein: NP_068761.4:p.?  Zygosity: Homozygous  Pathogenic | Known variant [87] | Muscular dystrophy, limb-girdle, autosomal recessive 18 (OMIM: 615356), autosomal recessive |
| ROU52 | 23/03/2019 | *PHF6* | Genomic Position: X-133527636-C-T (GRCh37)  DNA: NM_001015877.2:c.346C>T  Protein: NP_001015877.1:p.(Arg116Ter)  Zygosity: Heterozygous  Pathogenic | Known variant [88] | Borjeson-Forssman-Lehmann syndrome (OMIM: 301900), X-linked recessive |
| ROU53 | 14/08/2019 | *CPT1A* | Genomic Position: 11-68566685-C-T (GRCh37)  DNA: NM_001876.4:c.693+1G>A  Protein: NP_001867.2:p.?  Zygosity: Homozygous  Pathogenic | Known variant [89] | CPT deficiency, hepatic, type IA (OMIM: 255120), autosomal recessive |
| ROU54 | 19/08/2005 | *DPP6, PAXIP1 +21 more genes* | Genomic Position: NC_000007.13:g.(?_152055672)_(158937463_?)del (GRCh37)  Cytogenetic band: 7q35q36.3 (minimum size: 6.9Mb)  Type: Deletion  Zygosity: Heterozygous  Pathogenic | Known region [90, 91] | Distal monosomy 7q36 (ORPHA: 1636), autosomal dominant |
| ROU55 | 25/06/2015 | *SON* | Genomic Position: 21-34926431-TTAAC-T (GRCh37)  DNA: NM_138927.4:c.4897_4900del  Protein: NP_620305.3:p.(Thr1633Leu)fsTer9  Zygosity: Heterozygous  Likely Pathogenic | New variant | ZTTK syndrome (OMIM: 617140), autosomal dominant |
| ROU56 | 22/11/2020 | *CA2* | Genomic Position: 8-86385964-A-C (GRCh37)  DNA: NM_000067.3:c.275A>C  Protein: NP_000058.1:p.(Gln92Pro)  Zygosity: Homozygous  Likely Pathogenic | Known variant [92] | Osteopetrosis, autosomal recessive 3, with renal tubular acidosis (OMIM: 259730), autosomal recessive |
| ROU57 | 14/03/2022 | *ZBTB20* | Genomic Position: 3-114058192-G-C (GRCh37)  DNA: NM_001348800.3:c.1886C>G  Protein: NP_001335729.1:p.(Thr629Arg)  Zygosity: Heterozygous  Likely Pathogenic | New variant | Primrose syndrome (OMIM: 259050), autosomal dominant |
| ROU58 | 23/09/2020 | *MAP1B* | Genomic Position: 5-71411593-C-T (GRCh37)  DNA: NM_005909.5:c.253C>T  Protein: NP_005900.2:p.(Arg85Ter)  Zygosity: Heterozygous  Likely Pathogenic | New variant | Periventricular nodular heterotopia 9 (OMIM: 618918), autosomal dominant |
| ROU59 | 27/07/2021 | *MECP2* | Genomic Position: X-153296225-T-TA (GRCh37)  DNA: NM_001110792.2:c.1089_1090insT  Protein: NP_001104262.1:p.(Lys364Ter)  Zygosity: Heterozygous  Likely Pathogenic | New variant | Rett syndrome (OMIM: 312750), X-linked dominant |
| ROU60 | 28/03/2020 | *EEF1A2* | Genomic Position: 20-62122064-C-T (GRCh37)  DNA: NM_001958.5:c.797G>A  Protein: NP_001949.1:p.(Arg266Gln)  Zygosity: Heterozygous  Likely Pathogenic | New variant | Intellectual developmental disorder, autosomal dominant 38 (OMIM: 616393), autosomal dominant |
| ROU61 | 21/08/2019 | *ARID2* | Genomic Position: 12-46123837-A-T (GRCh37)  DNA: NM_152641.4:c.103A>T  Protein: NP_689854.2:p.(Lys35Ter)  Zygosity: Heterozygous  Likely Pathogenic | Known variant [93] | Coffin-Siris syndrome 6 (OMIM: 617808), autosomal dominant |
| ROU62 | 12/12/2021 | *BBS12* | Genomic Position: 4-123664110-C-T (GRCh37)  DNA: NM_152618.3:c.1063C>T  Protein: NP_689831.2:p.(Arg355Ter)  Zygosity: Homozygous  Pathogenic | Known variant [94] | Bardet-Biedl syndrome 12 (OMIM: 615989), autosomal recessive |
| ROU63 | 30/01/2017 | *GAP43, LSAMP +56 more genes* | Genomic Position: NC_000003.11:g.(?_115342537)_(124215260_?)del (GRCh37)  Cytogenetic band: 3q13.31q21.2 (minimum size: 8.87Mb)  Type: Deletion  Zygosity: Heterozygous  Pathogenic | Known region [95] | Chromosome 3q13.31 deletion syndrome (OMIM: 615433), autosomal dominant |
| ROU64 | 24/12/2021 | *GOLGA8S, GOLGA6L2 +13 more genes* | Genomic Position: NC_000015.9:g.(?_23609490)_(28566579_?)del (GRCh37)  Cytogenetic band: 15q11.2q13.1 (minimum size: 4.95Mb)  Type: Deletion  Zygosity: Heterozygous  Pathogenic | Known region [96] | Chromosome 15q11.2q13.1 Deletion (OMIM: 105830 and OMIM: 176270), autosomal dominant |

**REFERENCES**

1. Laing, N.G., et al., *Mutations and polymorphisms of the skeletal muscle alpha-actin gene (ACTA1).* Hum Mutat, 2009. **30**(9): p. 1267-77.

2. Davis, M.R., et al., *Principal mutation hotspot for central core disease and related myopathies in the C-terminal transmembrane region of the RYR1 gene.* Neuromuscul Disord, 2003. **13**(2): p. 151-7.

3. Fernández-Marmiesse, A., et al., *Homozygous truncating mutation in prenatally expressed skeletal isoform of TTN gene results in arthrogryposis multiplex congenita and myopathy without cardiac involvement.* Neuromuscular Disorders, 2017. **27**(2): p. 188-192.

4. Wan, J., et al., *Mutations in the RNA exosome component gene EXOSC3 cause pontocerebellar hypoplasia and spinal motor neuron degeneration.* Nat Genet, 2012. **44**(6): p. 704-8.

5. Eggens, V.R., et al., *EXOSC3 mutations in pontocerebellar hypoplasia type 1: novel mutations and genotype-phenotype correlations.* Orphanet J Rare Dis, 2014. **9**: p. 23.

6. Kelberman, D., et al., *Mutations within Sox2/SOX2 are associated with abnormalities in the hypothalamo-pituitary-gonadal axis in mice and humans.* J Clin Invest, 2006. **116**(9): p. 2442-55.

7. Van de Vondel, L., et al., *De Novo and Dominantly Inherited SPTAN1 Mutations Cause Spastic Paraplegia and Cerebellar Ataxia.* Mov Disord, 2022. **37**(6): p. 1175-1186.

8. Godfrey, C., et al., *Refining genotype phenotype correlations in muscular dystrophies with defective glycosylation of dystroglycan.* Brain, 2007. **130**(Pt 10): p. 2725-35.

9. Quijano-Roy, S., et al., *De novo LMNA mutations cause a new form of congenital muscular dystrophy.* Ann Neurol, 2008. **64**(2): p. 177-86.

10. Pasqualin, L.M., et al., *Congenital muscular dystrophy with dropped head linked to the LMNA gene in a Brazilian cohort.* Pediatr Neurol, 2014. **50**(4): p. 400-6.

11. Züchner, S., et al., *Axonal neuropathy with optic atrophy is caused by mutations in mitofusin 2.* Ann Neurol, 2006. **59**(2): p. 276-81.

12. Di Meglio, C., et al., *Clinical and allelic heterogeneity in a pediatric cohort of 11 patients carrying MFN2 mutation.* Brain Dev, 2016. **38**(5): p. 498-506.

13. Casasnovas, C., et al., *Phenotypic spectrum of MFN2 mutations in the Spanish population.* J Med Genet, 2010. **47**(4): p. 249-56.

14. Brožková, D., et al., *Spectrum and frequencies of mutations in the MFN2 gene and its phenotypical expression in Czech hereditary motor and sensory neuropathy type II patients.* Mol Med Rep, 2013. **8**(6): p. 1779-84.

15. Laššuthová, P., et al., *Improving diagnosis of inherited peripheral neuropathies through gene panel analysis.* Orphanet J Rare Dis, 2016. **11**(1): p. 118.

16. Eisenberg, I., et al., *The UDP-N-acetylglucosamine 2-epimerase/N-acetylmannosamine kinase gene is mutated in recessive hereditary inclusion body myopathy.* Nat Genet, 2001. **29**(1): p. 83-7.

17. Richard, I., et al., *Multiple independent molecular etiology for limb-girdle muscular dystrophy type 2A patients from various geographical origins.* Am J Hum Genet, 1997. **60**(5): p. 1128-38.

18. Milic, A., et al., *A third of LGMD2A biopsies have normal calpain 3 proteolytic activity as determined by an in vitro assay.* Neuromuscul Disord, 2007. **17**(2): p. 148-56.

19. Mojbafan, M., et al., *Linkage Study Revealed Complex Haplotypes in a Multifamily due to Different Mutations in CAPN3 Gene in an Iranian Ethnic Group.* J Mol Neurosci, 2016. **59**(3): p. 392-6.

20. O'Grady, G.L., et al., *Variants in the Oxidoreductase PYROXD1 Cause Early-Onset Myopathy with Internalized Nuclei and Myofibrillar Disorganization.* Am J Hum Genet, 2016. **99**(5): p. 1086-1105.

21. Lamandé, S.R., et al., *Bethlem myopathy and engineered collagen VI triple helical deletions prevent intracellular multimer assembly and protein secretion.* J Biol Chem, 1999. **274**(31): p. 21817-22.

22. de Paula, F., et al., *Asymptomatic carriers for homozygous novel mutations in the FKRP gene: the other end of the spectrum.* Eur J Hum Genet, 2003. **11**(12): p. 923-30.

23. Babameto-Laku, A., et al., *The first case of primary alpha-sarcoglycanopathy identified in Albania, in two siblings with homozygous alpha-sarcoglycan mutation.* Genet Couns, 2011. **22**(4): p. 377-83.

24. Reddy, H.M., et al., *Homozygous nonsense mutation in SGCA is a common cause of limb-girdle muscular dystrophy in Assiut, Egypt.* Muscle Nerve, 2016. **54**(4): p. 690-5.

25. Bonne, G., et al., *Mutations in the gene encoding lamin A/C cause autosomal dominant Emery-Dreifuss muscular dystrophy.* Nat Genet, 1999. **21**(3): p. 285-8.

26. Mitsuhashi, H., et al., *Specific phosphorylation of Ser458 of A-type lamins in LMNA-associated myopathy patients.* J Cell Sci, 2010. **123**(Pt 22): p. 3893-900.

27. Magagnotti, C., et al., *Protein profiling reveals energy metabolism and cytoskeletal protein alterations in LMNA mutation carriers.* Biochim Biophys Acta, 2012. **1822**(6): p. 970-9.

28. Kajino, S., et al., *Congenital fiber type disproportion myopathy caused by LMNA mutations.* J Neurol Sci, 2014. **340**(1-2): p. 94-8.

29. Noguchi, S., et al., *Mutations in the dystrophin-associated protein gamma-sarcoglycan in chromosome 13 muscular dystrophy.* Science, 1995. **270**(5237): p. 819-22.

30. Richard, I., et al., *Mutations in the proteolytic enzyme calpain 3 cause limb-girdle muscular dystrophy type 2A.* Cell, 1995. **81**(1): p. 27-40.

31. Meyer-Kleine, C., et al., *Spectrum of mutations in the major human skeletal muscle chloride channel gene (CLCN1) leading to myotonia.* Am J Hum Genet, 1995. **57**(6): p. 1325-34.

32. Brugnoni, R., et al., *A large cohort of myotonia congenita probands: novel mutations and a high-frequency mutation region in exons 4 and 5 of the CLCN1 gene.* J Hum Genet, 2013. **58**(9): p. 581-7.

33. National Center for Biotechnology Information. ClinVar; [VCV000285648.11], <https://www.ncbi.nlm.nih.gov/clinvar/variation/VCV000285648.11> (accessed Sept. 12, 2024).

34. National Center for Biotechnology Information. ClinVar; [VCV000202405.12], <https://www.ncbi.nlm.nih.gov/clinvar/variation/VCV000202405.12> (accessed Sept. 12, 2024).

35. Cabrera-Serrano, M., et al., *Expanding the phenotype of GMPPB mutations.* Brain, 2015. **138**(Pt 4): p. 836-44.

36. Bergant, G., et al., *Comprehensive use of extended exome analysis improves diagnostic yield in rare disease: a retrospective survey in 1,059 cases.* Genet Med, 2018. **20**(3): p. 303-312.

37. Fecarotta, S., et al., *Steroid therapy in an alpha-dystroglycanopathy due to GMPPB gene mutations: A case report.* Neuromuscul Disord, 2018. **28**(11): p. 956-960.

38. Astrea, G., et al., *Broad phenotypic spectrum and genotype-phenotype correlations in GMPPB-related dystroglycanopathies: an Italian cross-sectional study.* Orphanet J Rare Dis, 2018. **13**(1): p. 170.

39. Cameron-Christie, S.R., et al., *Recessive Spondylocarpotarsal Synostosis Syndrome Due to Compound Heterozygosity for Variants in MYH3.* Am J Hum Genet, 2018. **102**(6): p. 1115-1125.

40. National Center for Biotechnology Information. ClinVar; [VCV000567557.6], <https://www.ncbi.nlm.nih.gov/clinvar/variation/VCV000567557.6> (accessed Sept. 13, 2024).

41. Jarraya, M., et al., *Whole-Body muscle MRI in a series of patients with congenital myopathy related to TPM2 gene mutations.* Neuromuscul Disord, 2012. **22 Suppl 2**: p. S137-47.

42. Mokbel, N., et al., *K7del is a common TPM2 gene mutation associated with nemaline myopathy and raised myofibre calcium sensitivity.* Brain, 2013. **136**(Pt 2): p. 494-507.

43. Davidson, A.E., et al., *Novel deletion of lysine 7 expands the clinical, histopathological and genetic spectrum of TPM2-related myopathies.* Brain, 2013. **136**(Pt 2): p. 508-21.

44. Nakamura, A., et al., *Deletion of exons 3-9 encompassing a mutational hot spot in the DMD gene presents an asymptomatic phenotype, indicating a target region for multiexon skipping therapy.* J Hum Genet, 2016. **61**(7): p. 663-7.

45. Sadikovic, B., et al., *Sequence homology at the breakpoint and clinical phenotype of mitochondrial DNA deletion syndromes.* PLoS One, 2010. **5**(12): p. e15687.

46. Jeppesen, T.D., et al., *Muscle phenotype and mutation load in 51 persons with the 3243A>G mitochondrial DNA mutation.* Arch Neurol, 2006. **63**(12): p. 1701-6.

47. Parsons, T., et al., *Autonomic symptoms in carriers of the m.3243A>G mitochondrial DNA mutation.* Arch Neurol, 2010. **67**(8): p. 976-9.

48. Malfatti, E., et al., *High risk of severe cardiac adverse events in patients with mitochondrial m.3243A>G mutation.* Neurology, 2013. **80**(1): p. 100-5.

49. Cataldo, L.R., et al., *[Mitochondrial DNA heteroplasmy of the m.3243A>G mutation in maternally inherited diabetes and deafness].* Rev Med Chil, 2013. **141**(3): p. 305-12.

50. Dvorakova, V., et al., *The phenotypic spectrum of fifty Czech m.3243A>G carriers.* Mol Genet Metab, 2016. **118**(4): p. 288-95.

51. Riley, L.G., et al., *The diagnostic utility of genome sequencing in a pediatric cohort with suspected mitochondrial disease.* Genet Med, 2020. **22**(7): p. 1254-1261.

52. Dawod, P.G.A., et al., *Mutational Analysis and mtDNA Haplogroup Characterization in Three Serbian Cases of Mitochondrial Encephalomyopathies and Literature Review.* Diagnostics (Basel), 2021. **11**(11).

53. Chung, C.Y., et al., *Constitutive activation of the PI3K-Akt-mTORC1 pathway sustains the m.3243 A > G mtDNA mutation.* Nat Commun, 2021. **12**(1): p. 6409.

54. Koshikawa, N., et al., *A linear five-ring pyrrole-imidazole polyamide-triphenylphosphonium conjugate targeting a mitochondrial DNA mutation efficiently induces apoptosis of HeLa cybrid cells carrying the mutation.* Biochem Biophys Res Commun, 2021. **576**: p. 93-99.

55. National Center for Biotechnology Information. ClinVar; [VCV002663477.1], <https://www.ncbi.nlm.nih.gov/clinvar/variation/VCV002663477.1> (accessed Jan. 22, 2025).

56. National Center for Biotechnology Information. ClinVar; [VCV002109465.2], <https://www.ncbi.nlm.nih.gov/clinvar/variation/VCV002109465.2> (accessed Sept. 12, 2024).

57. National Center for Biotechnology Information. ClinVar; [VCV000590572.15], <https://www.ncbi.nlm.nih.gov/clinvar/variation/VCV000590572.15> (accessed Sept. 12, 2024).

58. Frazier, T.W., et al., *Molecular and phenotypic abnormalities in individuals with germline heterozygous PTEN mutations and autism.* Mol Psychiatry, 2015. **20**(9): p. 1132-8.

59. MacLeod, H., et al., *A novel FKRP mutation in congenital muscular dystrophy disrupts the dystrophin glycoprotein complex.* Neuromuscul Disord, 2007. **17**(4): p. 285-9.

60. Scoto, M., et al., *Novel mutations expand the clinical spectrum of DYNC1H1-associated spinal muscular atrophy.* Neurology, 2015. **84**(7): p. 668-79.

61. Cossins, J., et al., *The spectrum of mutations that underlie the neuromuscular junction synaptopathy in DOK7 congenital myasthenic syndrome.* Hum Mol Genet, 2012. **21**(17): p. 3765-75.

62. Ziats, M.N., et al., *Genotype-phenotype analysis of 523 patients by genetics evaluation and clinical exome sequencing.* Pediatr Res, 2020. **87**(4): p. 735-739.

63. Selcen, D., et al., *Dok-7 myasthenia: phenotypic and molecular genetic studies in 16 patients.* Ann Neurol, 2008. **64**(1): p. 71-87.

64. Vytopil, M., et al., *Mutation analysis of the lamin A/C gene (LMNA) among patients with different cardiomuscular phenotypes.* J Med Genet, 2003. **40**(12): p. e132.

65. Rudenskaya, G.E., et al., *Laminopathies in Russian families.* Clin Genet, 2008. **74**(2): p. 127-33.

66. Verhoeven, K., et al., *Hearing impairment and neurological dysfunction associated with a mutation in the mitochondrial tRNASer(UCN) gene.* Eur J Hum Genet, 1999. **7**(1): p. 45-51.

67. Jacobs, H.T., et al., *Mitochondrial DNA mutations in patients with postlingual, nonsyndromic hearing impairment.* Eur J Hum Genet, 2005. **13**(1): p. 26-33.

68. Fetoni, V., et al., *Monomelic amyotrophy associated with the 7472insC mutation in the mtDNA tRNASer(UCN) gene.* Neuromuscul Disord, 2004. **14**(11): p. 723-6.

69. Lévêque, M., et al., *Whole mitochondrial genome screening in maternally inherited non-syndromic hearing impairment using a microarray resequencing mitochondrial DNA chip.* Eur J Hum Genet, 2007. **15**(11): p. 1145-55.

70. Ensink, R.J., et al., *Early-onset sensorineural hearing loss and late-onset neurologic complaints caused by a mitochondrial mutation at position 7472.* Arch Otolaryngol Head Neck Surg, 1998. **124**(8): p. 886-91.

71. Jaksch, M., et al., *Progressive myoclonus epilepsy and mitochondrial myopathy associated with mutations in the tRNA(Ser(UCN)) gene.* Ann Neurol, 1998. **44**(4): p. 635-40.

72. Délot, E., et al., *Trinucleotide expansion mutations in the cartilage oligomeric matrix protein (COMP) gene.* Hum Mol Genet, 1999. **8**(1): p. 123-8.

73. Li, L., et al., *Genotypic and phenotypic characterization of Chinese patients with osteogenesis imperfecta.* Hum Mutat, 2019. **40**(5): p. 588-600.

74. Clinical Genome Resource. <https://search.clinicalgenome.org/kb/gene-dosage/region/ISCA-37436#> (accessed Sept. 13, 2024).

75. National Center for Biotechnology Information. ClinVar; [VCV001180757.1], <https://www.ncbi.nlm.nih.gov/clinvar/variation/VCV001180757.1> (accessed Sept. 13, 2024).

76. Courtens, W., et al., *A subterminal deletion of the long arm of chromosome 10: a clinical report and review.* Am J Med Genet A, 2006. **140**(4): p. 402-9.

77. Yatsenko, S.A., et al., *Identification of critical regions for clinical features of distal 10q deletion syndrome.* Clin Genet, 2009. **76**(1): p. 54-62.

78. National Center for Biotechnology Information. ClinVar; [VCV001387304.5], <https://www.ncbi.nlm.nih.gov/clinvar/variation/VCV001387304.5> (accessed Jan. 22, 2025).

79. National Center for Biotechnology Information. ClinVar; [VCV000814334.1], <https://www.ncbi.nlm.nih.gov/clinvar/variation/VCV000814334.1> (accessed Sept. 13, 2024).

80. National Center for Biotechnology Information. ClinVar; [VCV000149685.2], <https://www.ncbi.nlm.nih.gov/clinvar/variation/VCV000149685.2> (accessed Sept. 13, 2024).

81. Taylor, C.M., et al., *16p11.2 Recurrent Deletion*, in *GeneReviews(®)*, M.P. Adam, et al., Editors. 1993, University of Washington, Seattle

Copyright © 1993-2024, University of Washington, Seattle. GeneReviews is a registered trademark of the University of Washington, Seattle. All rights reserved.: Seattle (WA).

82. Weiss, L.A., et al., *Association between microdeletion and microduplication at 16p11.2 and autism.* N Engl J Med, 2008. **358**(7): p. 667-75.

83. Cooper, G.M., et al., *A copy number variation morbidity map of developmental delay.* Nat Genet, 2011. **43**(9): p. 838-46.

84. Bin, J., et al., *BBS7 and TTC8 (BBS8) mutations play a minor role in the mutational load of Bardet-Biedl syndrome in a multiethnic population.* Hum Mutat, 2009. **30**(7): p. E737-46.

85. Leslie, N.D., et al., *The human galactose-1-phosphate uridyltransferase gene.* Genomics, 1992. **14**(2): p. 474-80.

86. Schadewaldt, P., et al., *Endogenous galactose formation in galactose-1-phosphate uridyltransferase deficiency.* Arch Physiol Biochem, 2014. **120**(5): p. 228-39.

87. Bögershausen, N., et al., *Recessive TRAPPC11 mutations cause a disease spectrum of limb girdle muscular dystrophy and myopathy with movement disorder and intellectual disability.* Am J Hum Genet, 2013. **93**(1): p. 181-90.

88. Gerber, C.B., et al., *Further characterization of Borjeson-Forssman-Lehmann syndrome in females due to de novo variants in PHF6.* Clin Genet, 2022. **102**(3): p. 182-190.

89. Yu, Y., et al., *[Clinical features and gene mutations of 6 patients with carnitine palmitoyltransferase 1A deficiency].* Zhonghua Yi Xue Za Zhi, 2021. **101**(14): p. 1041-1044.

90. Jackson, C.C., et al., *Kaposi sarcoma, oral malformations, mitral dysplasia, and scoliosis associated with 7q34-q36.3 heterozygous terminal deletion.* Am J Med Genet A, 2017. **173**(7): p. 1858-1865.

91. Fan, L.L., et al., *Case Report: Congenital Brain Dysplasia, Developmental Delay and Intellectual Disability in a Patient With a 7q35-7q36.3 Deletion.* Front Genet, 2021. **12**: p. 761003.

92. Hu, P.Y., et al., *Seven novel mutations in carbonic anhydrase II deficiency syndrome identified by SSCP and direct sequencing analysis.* Hum Mutat, 1997. **9**(5): p. 383-7.

93. National Center for Biotechnology Information. ClinVar; [VCV003347919.1], <https://www.ncbi.nlm.nih.gov/clinvar/variation/VCV003347919.1> (accessed Jan. 22, 2025).

94. Stoetzel, C., et al., *Identification of a novel BBS gene (BBS12) highlights the major role of a vertebrate-specific branch of chaperonin-related proteins in Bardet-Biedl syndrome.* Am J Hum Genet, 2007. **80**(1): p. 1-11.

95. Molin, A.M., et al., *A novel microdeletion syndrome at 3q13.31 characterised by developmental delay, postnatal overgrowth, hypoplastic male genitals, and characteristic facial features.* J Med Genet, 2012. **49**(2): p. 104-9.

96. Clinical Genome Resource. <https://search.clinicalgenome.org/kb/gene-dosage/region/ISCA-37404> (accessed Sept. 13, 2024).
